# Heterotypic signaling of cancer-associated fibroblasts in shaping the cancer cell drug resistance

**DOI:** 10.20517/cdr.2022.72

**Published:** 2023-03-27

**Authors:** Ramesh Butti, Ashwini Khaladkar, Priya Bhardwaj, Gopinath Prakasam

**Affiliations:** ^1^Kidney Cancer Program, Simmons Comprehensive Cancer Centre, University of Texas Southwestern Medical Center, Dallas, TX 75235, USA.; ^2^Department of Biosciences and Bioengineering, Indian Institute of Technology Bombay, Bombay 400076, India.; ^3^Department of Pharmacology, All India Institute of Medical Sciences, New Delhi 110029, India.; ^#^Authors contributed equally.

**Keywords:** Tumor microenvironment, CAFs, heterogeneity, ECM, metabolic reprogramming, heterotypic signaling, drug resistance, natural products

## Abstract

The context-dependent reciprocal interaction between the cancer cells and surrounding fibroblasts is imperative for regulating malignant potential, metabolic reprogramming, immunosuppression, and ECM deposition. However, recent evidence also suggests that cancer-associated fibroblasts induce chemoresistance in cancer cells to various anticancer regimens. Because of the protumorigenic function of cancer-associated fibroblasts, these stromal cell types have emerged as fascinating therapeutic targets for cancer. However, this notion was recently challenged by studies that targeted cancer-associated fibroblasts and highlighted the underlying heterogeneity by identifying a subset of these cells with tumor-restricting functions. Hence, it is imperative to understand the heterogeneity and heterotypic signaling of cancer-associated fibroblasts to target tumor-promoting signaling processes by sparing tumor-restricting ones. In this review, we discuss the heterogeneity and heterotypic signaling of cancer-associated fibroblasts in shaping drug resistance and also list the cancer-associated fibroblast-targeting therapeutics.

## INTRODUCTION

Accumulation of genetic or epigenetic aberration may be important for the transformation of normal epithelial cells but not sufficient to induce malignant potential. Context-dependent interaction between cancer cells and tumor microenvironment components is imperative for malignant progression^[1]^. Tumor microenvironments consist of various kinds of non-cancerous cells such as fibroblasts, macrophages, mesenchymal stem cells (MSCs), pericytes, endothelial and immune cells, and extracellular matrix (ECM) known as tumor stroma^[2]^. Fibroblasts constitute a major component of tumor stroma and exhibit multipronged functions in tumor progression^[3,4]^.

Fibroblasts could be considered cockroaches of the human body as they thrive under severe stress and can even be isolated from decaying/dead tissue. Fibroblasts are quiescent cell types and synthetically and metabolically less active^[3]^. Upon activation, fibroblasts play a critical role in the wound healing process by remodeling ECM as well as secreting various growth factors and chemoattractant cytokines which ultimately regulate epithelial proliferation and immune cell infiltration^[3,5,6]^. Dysregulation of their activation leads to the formation of scar and fibrotic diseases. Fibroblasts associated with cancer, termed cancer-associated fibroblasts (CAFs), show functional and molecular differences from normal fibroblasts. Fibroblast activation by the secreted factors, cell-matrix or cell-cell contacts with cancer or other stromal cells leads to the CAF phenotype acquisition^[2,7-9]^. CAFs have been reported to exhibit higher migratory and contraction potentials along with synthesizing and remodeling ECM, reminiscent of myofibroblasts^[8,9]^. Several reports show that CAFs secrete a myriad of growth factors and cytokines which are critical for several facets of tumor progression. CAFs were known to regulate several hallmarks of cancer by directly influencing cancer cell proliferation, migration, invasion, and angiogenesis^[7-9]^. Our earlier study also reported that osteopontin (OPN)-activated CAF-derived CXCL12 promotes epithelial-to-mesenchymal transition (EMT) in breast cancer cells^[8]^. Moreover, CAFs are known to shape the tumor immune microenvironment through the elevated expression of immunosuppressive cytokines and immune checkpoint proteins that results in immunosuppression and tumor progression^[10]^. More importantly, CAFs are reported to induce drug resistance and cancer relapse in different cancers by different mechanisms including the induction of EMT, activation of stemness pathways, ECM remodeling, and dysregulated metabolism^[11]^. Due to their important functions in tumor progression, CAFs have emerged as an intriguing therapeutic target for the clinical control of cancer. However, the studies focused on targeting CAFs for the management of cancer have challenged this dogma. Of note, genetic ablation of the CAF population or fibrosis induces immunosuppressive environment in pancreatic ductal adenocarcinoma (PDAC) which in turn promotes EMT and invasion in cancer cells, leading to tumor progression with poor disease outcomes^[12]^. In addition, targeting the hedgehog (Hh) pathway in CAFs led to more aggressive and poorly differentiated PDAC with reduced stromal content and survival^[13,14]^. The above report highlights the presence of a subset of CAFs with tumor-restricting functions. Understanding the heterogeneity of CAFs and their heterotypic signaling might help in tailoring therapeutic intervention that selectively targets tumor-promoting CAF population and spares tumor-restraining ones. This review focuses on CAF heterogeneity and heterotypic signaling in regulating drug resistance to cancer therapies. This review also highlights several current CAF-targeted therapies for the treatment of different cancer types.

## NORMAL FIBROBLASTS AND ACTIVATED/CANCER-ASSOCIATED FIBROBLASTS

During the generation of the third germ layer or mesoderm, primitive mesenchymal cells (primary mesenchyme) first appear when the epiblast undergoes EMT^[15]^. Most of the active mesenchymal cells undergo apoptosis after the completion of tissue development, whereas few cells attain a quiescent phenotype, which was first observed by Virchow^[16] ^and eventually named fibroblasts. Normal fibroblasts are elongated cells with extended cell processes that exhibit a fusiform or spindle-like shape. These are generally present in connected tissues where they are embedded within ECM which consists largely of type I collagen and fibronectin^[17]^. A specific marker of quiescent fibroblasts is still missing; however, fibroblast-specific protein 1 (FSP1) and vimentin are considered as the closest. Normal fibroblasts also express integrins which are the mediators of the interaction of fibroblasts with their surrounding microenvironment^[17]^. Additionally, normal fibroblasts are characterized by low metabolic activity and lack of mobility^[3]^.

Fibroblasts can be activated to acquire activated/myofibroblast phenotype, which is associated with enhanced proliferative activity and increased synthesis of ECM proteins such as type I collagen, tenascin C, extra domain A (EDA)-splice variant of fibronectin, and secreted protein acidic and rich in cysteine (SPARC)^[17]^. Fibroblast activation can be promoted by various stimuli generated from tissue injury or damage, including transforming growth factor beta (TGF-β), epidermal growth factor (EGF), fibroblast growth factor 2 (FGF2), and interferon-γ (IFNγ), interleukin (IL-6), mechano-transductions and enzymes^[17-19]^. Upon activation, these cells exhibit prolific protein synthesis and higher contraction potential that is crucial for wound healing and the production of connective tissues^[3]^. In physiological conditions, myofibroblasts play a critical role in wound healing and repairing damaged tissues^[19-22]^. Upon the completion of their function, these cells are cleared by programmed cell death, apoptosis^[23]^. However, if the injury is perpetual or dysregulation of the cell death program of these cells, it can lead to hyperproliferation and accumulation of myofibroblasts which culminates in a condition known as fibrosis^[24-26]^.

“Tumors are depicted as wounds that do not heal” as they undergo continuous stromal remodeling and vascular growth, reminiscent of the wound repair program. Similar to wound healing process, activated fibroblasts/myofibroblasts are also present in tumors and are known as CAFs^[9]^. A diverse set of tumor or stroma-derived factors, including TGF-β1, OPN, and IL-1β, drive the transition of resting fibroblasts to CAFs by regulating Akt, ERK, MAPK, SMAD and NF-κB signaling pathways^[8,27-29]^. In an activation state, CAFs attain increased contractibility features and migratory potentials, which enables the CAFs to remodel ECM and aid in reciprocal interaction with cancer cells^[3,30,31]^. Different CAF-specific markers were identified to characterize activated CAFs, such as alpha-smooth muscle actin (α-SMA), fibroblast activation protein (FAP), FSP1 (also known as S100A4), Integrin β1 (CD29), platelet-derived growth factor receptor α or β (PDGFRα/β) or podoplanin (PDPN)^[32]^. PDGFRs are a class of RTKs, known to be involved in tumor-fibroblast interactions^[33]^. In contrast to wound healing, but similar to organ fibrosis, the fibroblasts at the tumor site remain perpetually activated and form fibrous growth in the tumor, referred to as desmoplastic reaction/stroma^[34]^. Moreover, it was observed that senescent fibroblasts, which resemble myofibroblasts, also support preneoplastic tumor growth via secretion of OPN^[35,36]^.

## ORIGIN OF CAFS

**Figure 1:**
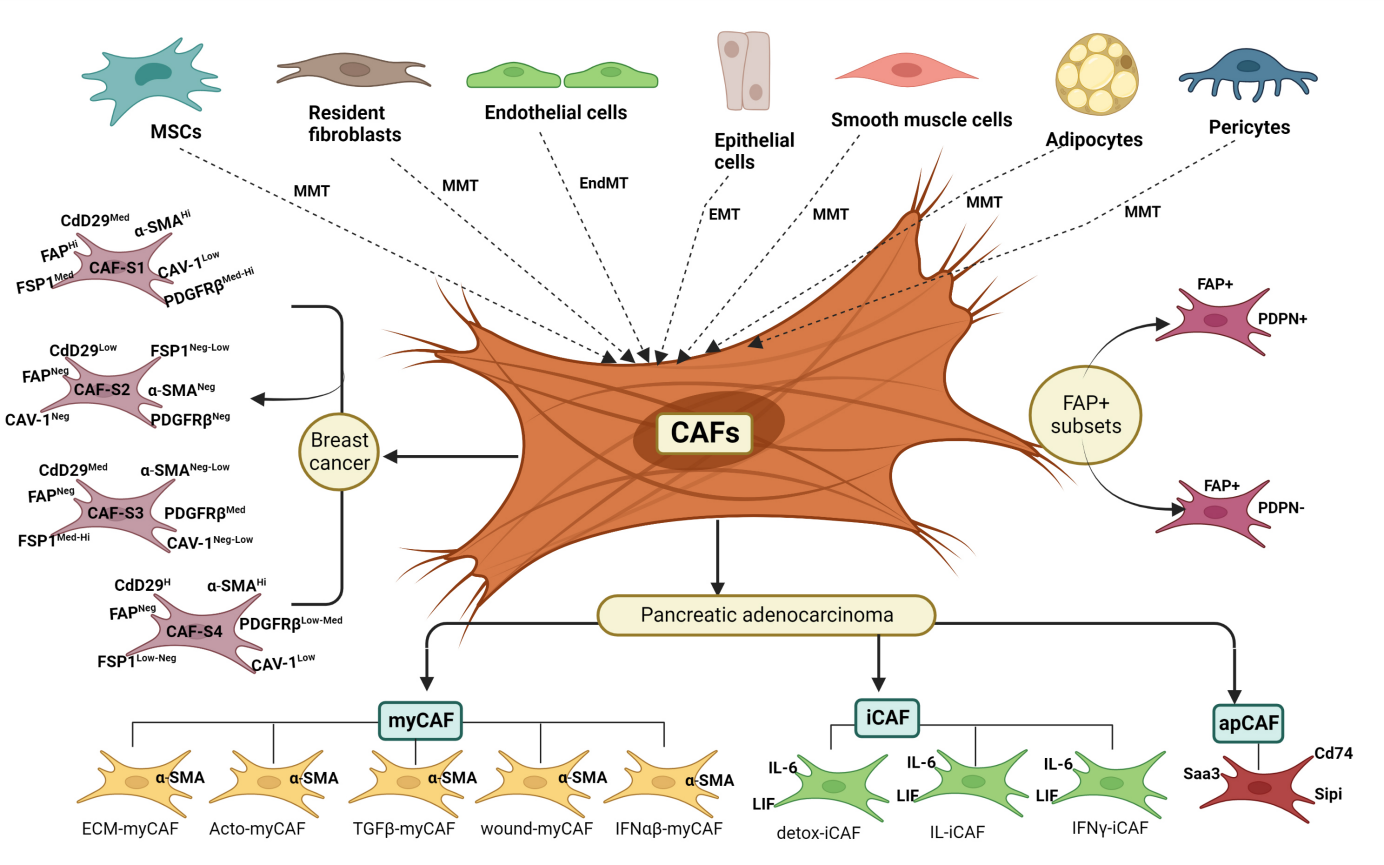
Origin and heterogeneity of cancer-associated fibroblasts. CAFs in the tumor microenvironment can be originated from MSCs, fibroblasts, adipocytes, pericytes, smooth muscle, endothelial and epithelial cells through the different trans-differentiation programs. Varieties of CAF subsets have been identified in cancer types of different tissue origins. The different subsets of CAFs show different functions and molecular features. CAF-S1 to CAF-S4 subsets are present in breast cancer. myCAF, iCAF and apCAF subsets are observed in PDAC. Several cancers exhibit overlapping populations of CAF subsets.

The expression of different kinds of markers in CAFs indicates the heterogeneous generation and different cellular sources of these cells. CAFs can be originated from epithelial cells through the EMT [Figure 1]. According to a report, epithelial cells undergo specialized EMT by MMP-driven oxidative stress-associated DNA oxidation and mutations that lead to transdifferentiation of these cells into activated myofibroblasts^[17,37]^. This hypothesis is mainly supported by genetic studies conducted on breast cancers. These studies have reported somatic mutations in TP53 and phosphatase and tensin homolog (PTEN), and gene copy number alteration at other loci in stromal CAFs, similar to mutations in epithelial cells. Moreover, p53 inactivation in stromal fibroblasts and genetic inactivation of PTEN in CAFs promote tumor progression in breast carcinoma models^[38-40]^. Collectively, these data indicate that the tumor-promoting activity of CAFs may depend on somatic mutations in these tumor suppressor genes. In addition, somatic alterations were frequently detected (> 30%) in tumor cell-surrounding fibroblasts^[39,40]^. Similarly, CAFs might be generated from cancerous epithelial cells by EMT [Figure 1]^[41]^. The EMT renders cancer cells to acquire mesenchymal phenotype and higher migration and contraction potentials^[15]^. This EMT program induced by platelet-derived growth factor (PDGF), TGFβ, EGF, etc., and is facilitated by the activation of mesenchymal specific transcription factors like Snail, Slug, Twist and FOXC2^[15,42]^. Tumor-associated endothelial cells might contribute to the CAF population [Figure 1]. A previous study has shown that endothelial cells are transdifferentiated into CAFs via endothelial to mesenchymal transition (EndMT) by losing expression of CD31 and gaining the expression of FSP-1 and α-SMA under the TGF-β stimulus^[43]^. In another study, auto/paracrine FGF2 has been shown to regulate the TGF-β-induced EndMT in tumor endothelial cells (TECs) via Elk1^[44]^. In a similar way, pericytes undergo pericytes to myofibroblast transition (PMT), a mesenchymal-to-mesenchymal transdifferentiation (MMT) process to generate CAFs in a microenvironment [Figure 1]. Hosaka *et al.* have recently reported that vascular pericytes are converted to CAFs by PDGF-BB to promote tumor growth and metastasis. PDGF-BB binds to PDGFRβ to induce the PMT program in pericytes^[45]^. CAFs are known to be generated from bone marrow-derived mesenchymal stem cells (MSCs) [Figure 1]^[46,47]^. Recruitment of MSCs takes place in many pathological conditions such as tissue repair, inflammation, and neoplasia. MSCs are recruited from the bone marrow into the tumors and subjected to activation similar to many inflammatory cells by a plethora of cytokines and growth factors derived from tumor cells or activated stroma^[46-48]^. The cytokines involved in the activation are vascular endothelial growth factor (VEGF), hepatocyte growth factor (HGF), basic fibroblast growth factor (bFGF), PDGF, EGF, CCL2, etc^[49-51]^. The previous report showed that labeled MSCs have been shown to localize tumor mass and thus differentiate into pericytes and CAFs by acquiring de novo expression of characteristic markers such as α-SMA, FAP, tenascin-c and thrombospondin1^[52]^. Reports have shown that tumor-derived OPN induces MSC transformation into CAFs via MZF1-mediated TGF-β expression to promote more aggressive local tumor growth and metastasis in breast cancer^[53]^. Even though different cell types contribute to CAFs, the major source of CAFs is resident fibroblasts. Resident fibroblasts in tumors undergo fibroblast to myofibroblast transition (FMT), a process of MMT to generate CAFs [Figure 1]^[18]^. Various growth factors and cytokines, mechanical forces and cell-cell contacts regulate the FMT process^[8,19,30]^. Evaluation of the FMT process in the tumor microenvironment was initially achieved by Kojima *et al.* in the fibroblast and cancer cell co-implantation xenograft model. Their studies have revealed that autocrine activation of TGF-β and SDF-1 (CXCL12) signaling leads to the acquisition of myofibroblast phenotype in fibroblasts^[54]^. CBL (CBF1, Suppressor of Hairless, Lag-1) and p53 are considered tumor suppressors in different cancers. Silencing of CBL and p53 in fibroblasts leads to the attainment of CAF phenotype in normal fibroblasts^[55]^. Shimoda *et al.* have reported that TIMPless fibroblasts reflects the traits of CAFs. According to their studies, deletion of TIMP instigates the expression of α-SMA in fibroblasts and increases the migration and contraction potentials^[56]^. A recent study has described the role of nodal in the conversion of normal ﬁbroblasts to CAFs with snail and TGF-β signaling pathway activation^[57]^. In addition, paracrine signaling cues derived from tumor cells play a major role in the acquisition of the CAF phenotype. Tumor-derived TGF-β is known to play a pivotal role in the generation of CAFs from the activation of resident fibroblasts^[58]^. Recruitment of fibroblasts into the tumor microenvironment is a prerequisite for CAF generation. A previous study has identified that tumor cell-derived Wnt7a recruits and activates ﬁbroblasts to CAFs to promote tumor aggressiveness. Wnt7a exhibits a fibroblast-activating role by potentiating TGF-β receptor signaling and not relying on canonical Wnt signaling^[59]^. Epigenetic switch involving p300-mediated STAT3 acetylation induces the ﬁbroblast activation to CAFs to support tumor invasion^[60]^. Mechanical forces and matrix stiffness induce several signaling pathways in the tumor microenvironment that are imperative for tumor aggressiveness. Matrix stiffness elevates the activity of Yes-associated protein (YAP) in nearby fibroblasts, thereby inducing the CAF phenotype in these cells^[19]^. Activated fibroblasts are reported to secrete various growth factors, cytokines such as SDF-1, IL-6, CXCL14, CCL5 and CCL7 and proteases such as MMP-2, MMP-9 and uPA to promote EMT in cancer^[18]^.

## HETEROGENEITY OF CAFS

The multipronged actions of CAFs on tumor cells probably reflect their heterogeneous population with context-dependent functions. Although CAFs are known to originate from resident fibroblasts, MSCs, endothelial cells, pericytes, epithelial cells, and adipocytes through trans-differentiation programs, CAF subsets have been represented as distinct cellular states rather than indicating their different cell origins. Costa *et al.* have identified four subsets of CAFs (CAF-S1, CAF-S2, CAF-S3 and CAF-S4) in breast cancer by combining the analysis of six CAF markers [Figure 1]. Higher levels of both CAF-S1 (FAP^High^ CD29^Med^ SMA^Med-High^ FSP1^Med^ PDGFRβ^Med-High ^CAV1^Low^) and CAF-S4 (FAP^Neg-Low^ CD29^High^ SMA^High^ FSP1^Low-Med^ PDGFRβ^Low-Med ^CAV1^Low^) subsets are reported in aggressive Her2+ and triple-negative breast cancer (TNBC)^[61]^. Moreover, accumulation of FAP^High^ CAF-S1 subset in early luminal breast cancers is associated with distant relapse^[62]^. In contrast, the CAF-S2 subset (CD29^Low^ FAP^Neg^ FSP1^Neg-Low ^α-SMA^Neg ^PDGFRβ^Neg^ CAV1^Neg^) is highly accumulated in the luminal breast cancer subtype whereas CAF-S3 fibroblasts (CD29^Med^ FAP^Neg^ FSP1^Med-High^ α-SMA^Neg^ PDGFRβ^Med^ CAV1^Low^) is observed in healthy tissues^[61-63]^. In addition, CAF-A (ECM remodeling) and CAF-B (myofibroblastic genes) are observed in colorectal cancer (CRC)^[64]^.

Givel *et al.* have demonstrated fibroblast heterogeneity in high-grade serous ovarian cancers (HGSOC) by defining four subsets of CAFs (CAF-S1 to S4) as described in breast cancer^[65]^. Mesenchymal HGSOC consists of high CAF-S1 fibroblasts, which modulate immunosuppressive functions by increasing infiltration, survival, and differentiation of CD25^+^FOXP3^+ ^T lymphocytes. SDF-1 β (CXCL12β) is specifically accumulated in the immunosuppressive CAF-S1 subset. Thus, their data highlight a CXCL12β-regulated stromal heterogeneity and immunosuppression in mesenchymal HGSOC^[65]^. The existence of CAF-S1 and CAF-S4 molecular signatures has been validated in lung cancer^[66] ^and head and neck cancer by leveraging publicly available single-cell data^[67]^. The presence of these two major CAF-S1/CAF-S4 myofibroblastic subpopulations was validated in different cancer types^[68]^. These data suggest the existence of both CAF-S1 and CAF-S4 myofibroblastic cells in distinct cancer types and across species.

Two subsets of CAFs were recently reported in pancreatic adenocarcinoma. One subset displays a matrix-synthesizing myofibroblastic phenotype termed myCAF, whereas another exhibits an immunomodulatory phenotype, inflammatory CAFs named iCAF [Figure 1] The CAFs proximal to the cancer cells show a myCAF phenotype with higher expression of α-SMA. Distal CAFs from the cancer cells express high levels of proinflammatory cytokines such as IL-6, G-CSF, CXCL1, and LIF and are defined as iCAFs^[69]^. IL-1 signaling induces iCAF signature, while TGF-β signaling controls myCAF signature by antagonizing the iCAF phenotype. Another study has demonstrated the two different subpopulations of CAF, named myofibroblastic CAFs (myCAFs) and inflammatory CAFs (iCAFs), by employing a 3D co-culture system of PDAC *in vitro*^[70]^. Further, Elyada *et al.* reported the third subtype of CAFs, named antigen-presenting CAFs (apCAFs), using single-cell RNA sequencing (scRNA-seq) in PDAC tissues, and these are characterized by expression of H2-Aa, H2-Ab1 (encoding α, β -chains of MHC II), CD74, secretory leukocyte peptidase inhibitor (SLPI) and serum amyloid A3 (Saa3) genes [Figure 1]. Also, apCAFs possess antioxidant response and are regulated by IFN-γ signaling *in vivo*^[71]^. Furthermore, other studies also confirmed the apCAF classification based on the results obtained using scRNA-seq in pancreatic cancer^[72,73]^. In addition, transcriptomics study in normal pancreatic cells of KPP mice revealed that cells that express mesothelial signature also show the expression of MHC II genes, implicating that apCAF could be of mesothelial origin^[74]^. Later, apCAFs subtype has also been reported in breast and lung cancer^[75-78]^. Interestingly, apCAFs activate the CD4 + T lymphocytes, which implies that CAFs have antigen-presenting properties similar to other antigen-presenting cells such as macrophages, dendritic cells and B cells immunomodulatory functions. However,the study on orthotopic murine models of lung cancer showed that lung apCAFs are tumor-suppressive cells^[77]^. Another report has revealed the presence of two FAP+ subsets on the basis of PDPN expression [Figure 1]^[79]^. The FAP^+^ PDPN^+^ fibroblasts show elevated expression of TGF-β signaling proteins and fibrosis-associated genes, whereas FAP^+^ PDPN^-^ cells displayed less expression of the same genes^[68,71,79]^. Moreover, a recent study further classified FAP^High^ CAFs into eight different clusters. Out of these clusters, five clusters (ECM-myCAF, Acto-myCAF, TGFβ-myCAF, wound-myCAF and IFNαβ-myCAF) belong to the myCAF subgroup and three clusters (detox-iCAF, IL-iCAF, IFNγ-iCAF) fall into the iCAF subgroup^[68]^. Therefore, CAFs possess multifaceted functions including tumor promotion and prevention based on the gene expression signatures.

## CAFS REGULATE DRUG RESISTANCE BY MODULATING CANCER CELL SURVIVAL

**Figure 2:**
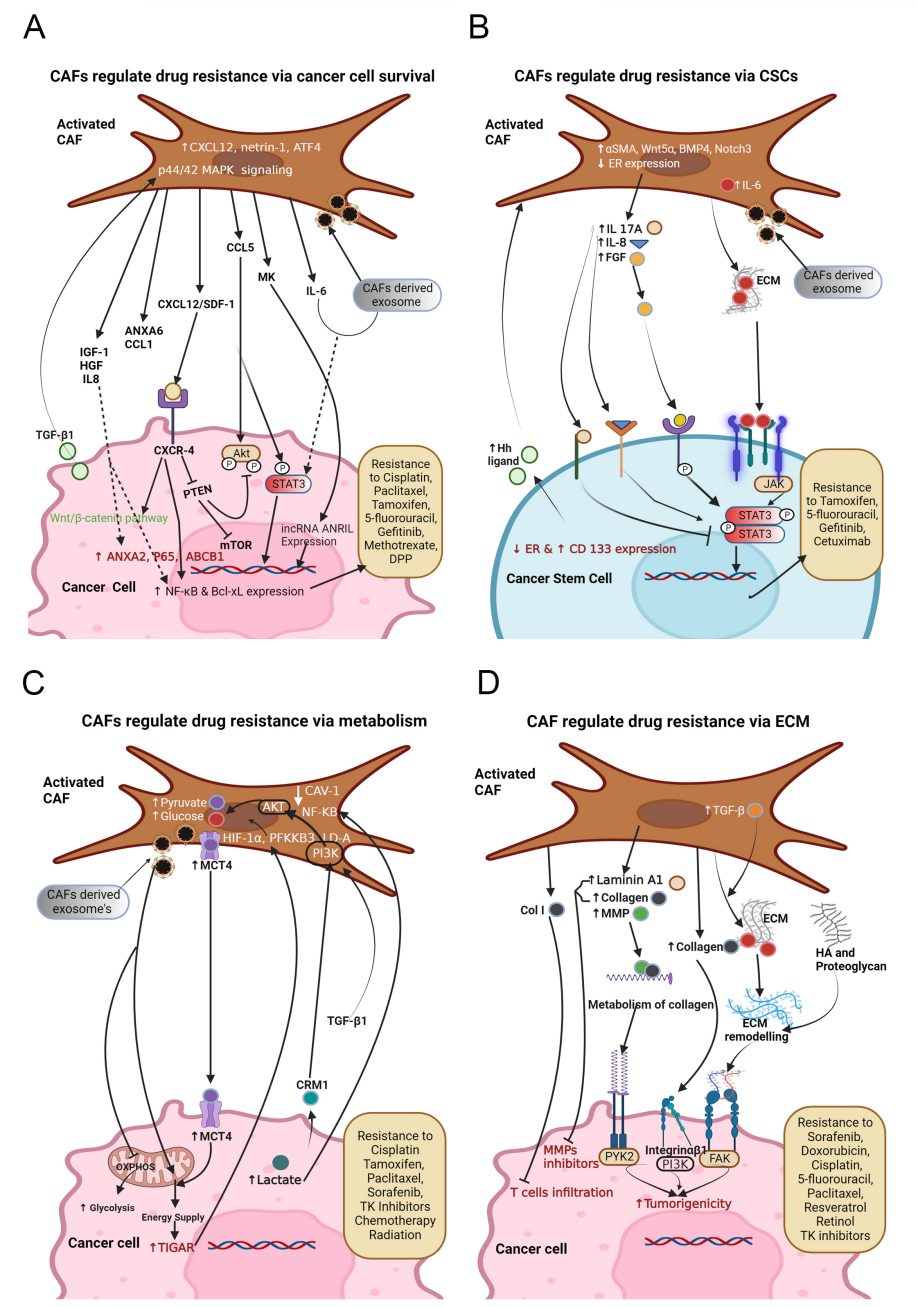
Fibroblast-mediated signaling in shaping the drug resistance in (A) CAFs promote cancer cells survival by secreting ANXA6, CCL1, CXCL 12, CCL5, MK, etc., and the exosomes containing lncRNA ANRIL, miR-196a, miR-103a-3p, miR-24-3p, microRNA-21, microRNA-148b-3p, LPP, CCAL, etc. Also, CAFs show high levels of netrin1, IL-6, and ATF4, which offer cancer cells gemcitabine resistance. TGF-β1 secreted by cancer cells acts on CAFs to attain 5-fluorouracil (5-FU) and tamoxifen (TAM) resistance. Inhibition of PTEN by CXCL12-CXCR4 binding promotes mTOR signaling and cancer proliferation. MK provides cisplatin resistance by ameliorating the expression of lncRNA-ANRIL; (B) CAFs induce stemness in cancer cells through the activation of STAT3 by IL8, IL6, and FGF and secreting exosomes containing miR-221 and H19 that leads to drug resistance, and STAT3 activation can be inhibited by IL-17A. High levels of α-SMA, Wnt5α, BMP4 and Notch3 in CAFs and low expression of ER in both CAFs and cancer cells are associated with enriching CSCs and drug resistance; (C) CAFs provide different nutrients to cancer cells. CAF-secreted factors rewire the cancer cell metabolism by the activation of autophagy, mTOR, and TIGAR and suppression of oxidative phosphorylation that leads to drug resistance; (D) CAFs promote drug resistance by ECM deposition. Activation of CAFs by TGF-β1 or other factors leads to excessive synthesis of ECM proteins such as laminin-A, collagen, and fibronectin. It also induces various MMPs, which leads to ECM modeling that blocks drug effects.

Interestingly, cancer cells produce a variety of factors that recruit, activate, and help with the survival of CAFs; nonetheless, CAFs, in return, support cancer cell survival and proliferation by providing appropriate signaling factors which subsequently promote cancer cell resistance. Using mouse models of inflammation-induced gastric cancer, a study reported that at least 20% of CAFs are derived from MSCs of bone marrow, and show the expression of α-SMA, wingless-related integration site 5α (Wnt5α), IL-6, bone morphogenetic protein 4 (BMP4), and DNA hypomethylation. MSC-derived CAFs are recruited to dysplastic stomach in TGF-β and SDF-1α-dependent manner to promote tumor survival^[80]^. CAFs provide ovarian cancer cells resistance to cisplatin by secreting cisplatin-induced chemokine (C-C motif) ligand 5 (CCL5), which augments the phosphorylation of STAT3 and Akt in cancer cells. Thus, CAFs play a crucial role in promoting ovarian cancer cell growth by regulating STAT3/PI3K/Akt pathway [Figure 2A]^[81]^. Interestingly, a heparin-binding growth factor, midkine (MK), derived from CAFs, provides cisplatin resistance to oral squamous cell carcinoma (OSCC), lung cancer, and ovarian cancer cells by enhancing the expression levels of the lncRNA-ANRIL [Figure 2A]. Therefore, targeting either MK production from CAFs or inhibiting the lncRNA-ANRIL in cancer cells could be the key to cancer treatment^[82]^. The mir-1-mediated expression of SDF-1 in CAFs induces the proliferation of lung cancer cells and chemoresistance via CXCR4-dependent pathway involving NF-κB and Bcl-xL^[83]^. CAFs are not only involved in promoting cancer cell viability but also induce EMT in response to drug treatments. An earlier study showed that CAFs promote EMT through the secretion of IGF-1 and HGF. These growth factors enhance the expression and phosphorylation of annexin A2 (ANXA2), which endorse the resistance to the EGFR-TKI (gefitinib) in NSCLC (HCC827 and PC9) cells-harboring EGFR activating mutations [Figure 2A]. Therefore, restricting the CAFs-induced EMT is necessary to subdue TKI-resistance^[84]^. EMT transcription factors such as Twist1 and Snail regulate the activation of CAFs in cancers^[30,85]^. Expression of Twist1 and Snail in CAFs also associated with the expression of several cytokines including SDF-1, CXCL1 and CCL2 which can regulate cell proliferation and survival^[30,86]^. Blanco-Gomez *et al.* demonstrated that loss of SNAI2 in CAFs limit the production of some cytokines such as SDF-1 and CXCL1, CXCL2, IFN-γ and IL-16, thereby impeding breast cancer cell proliferation and metastasis^[86]^. Thus, SNAI2 could be considered a therapeutic target to block both proliferation and EMT in tumor cells and cytokine production in CAFs.

Furthermore, CAFs elicit TGF-β-mediated EMT in ovarian cancer cells via IL-6-regulated JAK2/STAT3 pathway to inhibit cancer cell apoptosis and provide paclitaxel resistance^[87]^. CAF-secreted SDF-1 stimulates pancreatic cancer progression and aids in gemcitabine resistance by augmenting the expression of SATB-1^[88]^. A recent study showed that snail-positive fibroblasts facilitate chemoresistance to 5-fluorouracil and paclitaxel in colorectal cancer (CRC) by secreting CCL1 through the TGF-β/NF-κB signaling pathway^[89]^. In addition, CAFs upregulate the expression of the lipoma-preferred partner (LPP) in microvascular endothelial cells (MECs). The upregulated LPP upshots stress fiber formation and focal adhesion to further enhance the mobility and permeability of endothelial cells, which ultimately resulted in the enhancement of chemoresistance in ovarian cancer^[90]^. CAFs elevate human gastric cancer chemoresistance by higher expression of IL-8, which further regulates cell survival pathways including PI3K, Akt, IKb, p65, and ABCB1. Hence, IL-8 derived from CAFs involved in promoting chemoresistance in gastric cancer through NF-κB activation and upregulation of ABCB1 [Figure 2A]^[91]^. Annexin A6 present in CAFs-derived extracellular vesicles plays an important role in inducing drug resistance and tubular network formation in gastric cancer by activation of FAK/YAP axis through the stabilization of β1 integrin on the surface of cancer cells^[92]^. Higher expression of activating transcription factor 4 (ATF4) in PDAC-derived CAFs promotes malignancy and gemcitabine resistance through TGF-β1/SMAD2/3 axis^[93]^. Intriguingly, TGF-β1 secreted from breast cancer cells activates CAFs in a paracrine manner, contributing to chemoresistance via activating p44/42 MAPK signaling pathway^[94]^.

Higher expression of CXCL12 in interstitial CAFs contributes to EMT and cisplatin resistance in epithelial ovarian cancer (EOC) via CXCR4/Wnt/β-catenin pathway^[95]^. Additionally, this CAF-derived CXCL12 mediates inhibition of PTEN which is crucial for cancer cell proliferation [Figure 2A]^[96]^. Likewise, CAFs are involved in offering cisplatin resistance in HNC cells by exosome-mediated transfer of miR-196a. Upon depletion of CAF-exosomal miR-196a, restoration of cisplatin sensitivity has occurred in HNC cells. Therefore, targeting miR-196a can serve as a better therapeutic approach to overcome cisplatin resistance in HNC cells^[97]^. Moreover, CAF-derived, highly expressed, exosomal miR-103a-3p accelerates cisplatin resistance and inhibits apoptosis in NSCLC cells by targeting BCL2- antagonist/killer 1 (Bak1)^[98]^. CAF-derived miR-24-3p containing exosomes promote cancer cell resistance to methotrexate by downregulation of the CDX2/HEPH axis in colon cancer^[99]^. The CAF-mediated transfer of exosome-containing lncRNA CCAL (colorectal cancer-associated lncRNA) to CRC cells initiates signaling towards gaining resistance to oxaliplatin via the β-catenin pathway. Interaction of CCAL with HuR (human antigen R, an RNA stabilizing protein) leads to an increase in β-catenin, thereby providing oxaliplatin resistance in CRCs^[100]^. CAFs secreted IL-6/exosomal microRNA-21 (miR-21) induces the activation of STAT3 signaling to generate monocytic myeloid-derived suppressor cells (M-MDSCs) to further accelerate cisplatin (DDP). Therefore, inhibition of STAT3 signaling can restore cancer cells’ drug sensitivity^[101]^. Transfer of CAF derived exosomes-containing miR-148b-3p to bladder cancer cells enhances tumor proliferation, EMT, metastasis, and drug resistance. Mechanistically, miR-148b-3p induces Wnt/β-catenin pathway by targeting PTEN^[102]^. Therefore, overexpression of PTEN might lead to suppression of metastasis, EMT, and drug resistance. CAFs respond to tamoxifen treatment by upregulating the expression of high mobility group box 1 (HMGB1) through GPR30/PI3K/AKT signaling. HMGB1 is involved in the induction of autophagy to increase resistance to tamoxifen in MCF-7 cells via an ERK-mediated manner^[103]^. Overall, the above reports suggest that CAF-secreted growth factors, chemokines and exosomes regulate drug resistance by inducing cell survival in different types of cancer.

## CAFS REGULATE DRUG RESISTANCE BY MODULATING CANCER STEM CELLS

Cancer stem cells (CSCs) play a pivotal role in tumorigenesis, progression, and drug resistance. CSCs exhibit self-renewal and tumorigenic properties, which enable them to metastasize to distant sites, offering them a favorable environment. Moreover, the microenvironment around CSCs contributes a lot to fostering tumor growth through the modulation of CSC phenotype. The generation of CSCs through EMT is highly conditional on its surrounding matrix. This underscores the vital role of microenvironmental elements like CAFs and their secreted factors in shaping the renewal and maintenance of CSCs^[104]^.

Stem cell pathways like Wnt signaling are important for maintaining stemness in non-cancerous cells of the colon. An interesting study suggested that cells surrounding the CSCs, especially myofibroblasts, maintain a higher Wnt activity in CSCs and manage to stimulate Wnt signaling in nearby differentiated tumor cells, thereby mending the stemness and tumorigenicity^[105]^. In response to the chemotherapy, CAFs express IL-17A, which helps with the self-renewal of cancer-initiating cells (CICs) to facilitate resistance to chemotherapies^[106]^. Therefore, targeting IL-17A signaling could impede CICs growth. Additionally, exosomes secreted by fibroblasts in response to chemotherapy are also known to promote the spheregenerating capacity and chemotherapy resistance in CSCs. To validate the role of CAF-derived exosomes in priming CSCs, blockade of exosome release by culturing CAFs in the presence of a specific inhibitor of neutral sphingomyelinase 2, GW4869 resulted in the restoration of chemosensitivity in CSCs^[107]^. Hence, blocking CAFs secretion can be an effective approach to increasing the efficacy of chemotherapy in combating cancers. Specifically, fibroblast-derived exosomes-containing Wnts promote Wnt activity in CRC cells to enhance chemoresistance^[108]^. The microvesicles (MV) derived from CAF, transfer miR-221 to CSCs to induce hormonal therapy (HT)-resistance. The overall loop of events, including CAFs release of MV, is associated with a reduction of ER expression followed by an increase in Notch expression in CSCs. The increase in Notch further elicits the reduction of ER levels and an increase in CD133 levels in CSCs [Figure 2B]^[109]^. Moreover, CAFs are involved in supporting CSCs via combined activation of Wnt/β-catenin and HGF/Met signaling. CSCs regulate CAFs via secretion of Hh ligand, SHH to activate Hh signaling in a paracrine manner. In turn, CAFs secrete factors that help with CSC’s self-renewal and expansion. The treatment of tumors with a Hh inhibitor, vismodegib, led to the reduction of CAF activation and CSC’s expansion, thereby delaying the tumor formation and progression. Hence, targeting CAFs using Hh inhibitors can be an effective strategy for breast cancer treatment^[110]^. A study reported that Hh-stimulated CAFs contribute to the formation of chemo-resistant CSCs niche through the FGF pathway. Extracellular matrix rich in Hh-activated CAFs, FGF, and fibrillar collagen shape a conducive environment to foster a stem-like phenotype in triple-negative breast cancer (TNBC) cells^[111]^. Another study reported that HIF-1α and CAF-derived TGF-β2 crosstalk activate the expression of GLI2, a Hh transcription factor, in CSCs, which further enhances the chemoresistance and stemness of CSCs^[112]^. Specifically, CD10 and GPR77-positive subsets of CAFs are associated with chemoresistance by creating a niche for enrichment of CSCs in the multiple cohorts of breast and lung cancer patients. The binding of C5a to GPR77 in CD10 + GPR77 + CAFs in an autocrine manner phosphorylates p65 which ultimately leads to the expression of IL-6, IL-8, CD10, and GPR77. Though GPR77 continues to be part of autocrine cycle, IL-6 and IL-8 regulate CSC’s self-renewal^[113]^. Additionally, CAFs facilitate chemoresistance and stemness through the transfer of exosomal H19 to CRC. H19 produced by CAFs in CRC stroma mediates CSC phenotype by activating the β-catenin pathway^[114]^. The usage of MSC-derived fibroblasts (MSC-DF) has been reported for reciprocally studying the loss‐of‐function and gain‐of‐function of the Notch in the regulation of CSCs. It was found that MSC‐DF Notch1-/- promoted the formation of spheroids in co-cultured melanoma cells, while MSC‐DFN1IC+/+ (N1IC: Notch1 intracellular domain, an active form of Notch1) suppressed melanoma cell sphere formation capacity, thereby diminished tumor initiation properties. MSC‐DFNotch1-/- could contribute to promoting stemness of melanoma cells by upregulating Sox2/Oct4/Nanog expression^[115]^. An earlier report revealed that IL-17A acts as a CSC-maintenance factor and helps with CSC renewal and invasion^[116]^. Intriguingly, epigenetic changes in CAFs also play a critical role in maintaining CSC-promoting capacity. A recent study revealed that the loss of H3K27me3 in CAFs leads to the expression of WNT5A, NOG, GREM1, and IGF2, which play an important role in maintaining stem cell niche, stromal-epithelial interaction, and cell growth^[117]^. Therefore, it is important to identify players involved in CAF-specific epigenetic changes in order to shed light on epigenetic-centered CAF-targeted therapies. Netrin-1 is highly expressed in CSC and known to regulate stemness. The higher expression of netrin-1 was also found in CAFs and associated with the increasing stemness in cancer cells, thereby mediating drug resistance. Inhibition of intercellular signaling between cancer cells and CAFs using Netrin-1-mAb suppresses the expression of CAF-borne cytokines such as IL-6 ^[118]^. Taken together, CAF-regulated CSCs execute a crucial role in tumor initiation, maintenance, progression, and chemoresistance. Hence, finding a signaling messenger between CSCs and CAFs is indispensable for developing interventions to combat cancer

## CAFS REGULATE DRUG RESISTANCE BY MODULATING CANCER CELL METABOLISM

The major energy sources for the survival of unconditionally growing tumor cells are glutamine (Gln) and glucose. Rewiring of cancer cell metabolism enables the survival of cancer cells by providing the building blocks/intermediates for the synthesis of nucleic acids, lipids and proteins. Tumor microenvironment (TME) or CAFs-mediated metabolic reprogramming of cancer cells regulates several signaling cascades that also result in drug resistance^[119]^.

Notably, a study has shown that exosomes derived from CAFs can reprogram the metabolic machinery by their uptake into cancer cells^[120,121]^. These exosomes consist of intact lipids, amino acids, and intermediates of the TCA cycle^[121]^. Moreover, these exosomes inhibit mitochondrial OXPHOS, leading to increased glycolysis and Gln-dependent reductive carboxylation in cancer cells [Figure 2C]. It has been reported that CAFs predominantly express glucose uptake proteins in non-small cell lung cancer. Among these, glutamine-fructose-6-phosphate transaminase 2 (GFPT2) plays an important role in glycolysis, thus showing its significance in prognosis^[122]^. A previous study showed that epigenetic changes in CAF instigated a cascade of stromal-epithelial interactions to promote prostate cancer growth and resistance to androgen deprivation therapy (ADT). This study revealed that epigenetic silencing of a Ras inhibitor, RASAL3, in prostatic CAFs leads to oncogenic Ras activity that drives macropinocytosis-mediated glutamine synthesis. Interestingly, ADT further strengthens RASAL3 epigenetic silencing and glutamine secretion by CAFs. Therefore, high levels of glutamine have been found in prostate cancer patients after ADT^[123]^.

Cancer cells, under glucose-deprived states, use aerobic glycolysis as their major energy source, known as the Warburg effect. Pyruvate kinase M2 (PKM2) is overexpressed in NSCLC cell lines and plays a role in mediating the Warburg effect which promotes resistance to cisplatin^[124]^. In another phenomenon, aerobic glycolysis in the cancer-associated stroma metabolically supports surrounding cancer cells, which is known as the reverse Warburg effect. This stromal-cancer metabolic coupling enables catabolite transfer to cancer cells for the generation of ATP, induction of proliferation, and reduction of cell death^[125]^. Interestingly, cancer cells educate stromal cells to display aerobic glycolysis that mediates multidrug resistance^[126]^. Moreover, in the majority of solid tumors, CAFs utilize more glucose and in turn release more lactate in comparison to normal fibroblasts^[127]^. Notably, cancer cells induce the Warburg effect in CAFs through activation of the PI3K/AKT pathway via translocation of nuclear G-protein-coupled estrogen receptor (GPER) in a chromosomal region maintenance 1 (CRM1)-dependent manner and abnormal activation of the GPER/cAMP/PKA/CREB signaling pathway^[126]^. Consequently, CAFs delivered lactate transporters to cancer cells, which increases drug resistance [Figure 2C]. In contrast, a study by Apicella *et al.* showed that cancer cells expressing EGFR- or MET exhibit increased glycolytic activity leading to elevated levels of lactate. The elevated levels of lactate educate CAFs to secrete higher levels of HGF via an NF-κB-dependent mechanism that subsequently leads to cancer cell resistance against TKI therapy^[128]^. In solid tumors, the hypoxic environment mediates chemoresistance, as its low pH affects the cytotoxicity of mitoxantrone, paclitaxel and topotecan^[129]^. It has been reported that under a hypoxic environment, CAFs secrete several factors that activate angiogenic (VEGF) and immunogenic (T-cell mediated cytotoxicity) signaling that is essential for tumor progression^[130,131]^. A study showed that hypoxia induces migration, type I collagen expression, and VEGF production in pancreatic stellate cells and mediates resistance to anticancer drugs^[132]^. Interestingly, it has been demonstrated that TGF-β expressed by other stromal cells activates fibroblasts and induces ECM production, and stimulates aerobic glycolysis and catabolic metabolism^[133]^. It has been observed in a cancer cell-fibroblast co-culture system, oxidative stress-induced autophagy leads to downregulation of caveolin-1 (CAV1) in CAFs and overexpression of TIGAR (TP53- Induced Glycolysis and Apoptosis Regulator) in adjacent cancer cells^[134]^. Downregulation of CAV1 in CAFs leads to mitochondrial dysfunction and glycolysis via HIF-1α and NF-kB signaling. Therefore, autophagic CAFs prevent cancer cell death by providing substrates for metabolic activity of cancer cells and upregulating TIGAR which confers resistance to tamoxifen-induced apoptosis and autophagy^[135]^. In addition, overexpression of TIGAR in cancer cells induces a glycolytic phenotype in CAFs and promotes the expression of HIF-1 α, PFKFB3 (6-phosphofructo-2-kinase/fructose-2,6-biphosphatase 3) and lactate dehydrogenase-A along with increasing glucose uptake [Figure 2C]^[134]^. Overexpression of PFKFB3 has been shown to generate resistance to the BCR-ABL TKI in chronic myeloid leukemia (CML)^[136]^. Likewise, overexpression of HIF-1α along with glycolytic isoenzymes has been reported to be strongly associated with chemoresistance in different tumors^[137,138]^. Furthermore, CAFs-associated metabolic reprogramming also regulates epigenetic changes for the maintenance of the CAF active state; thereby, catabolic CAFs and anabolic cancer cells are metabolically coupled, contributing to the development of chemoresistant tumors^[139-141]^. In addition, the decrease in glucose concentration limits the synthesis of building blocks required for cell proliferation, leading to inhibition of cell proliferation. However, glucose starvation induces AMP-activated protein kinase (AMPK), which is an upstream factor of Hippo signaling; therefore, coupling of the metabolic pathway and Hippo signaling promotes drug resistance^[142]^. Metabolic coupling of CAFs and cancer cells is critical for regulating resistance to different therapeutic regimens. Therefore, devising therapeutic interventions to disrupt the metabolic coupling of CAFs and cancer may open new avenues for cancer treatment. However, how cancer cells educate CAFs to trigger resistance-mediating pathways is still poorly known.

## CAFS REGULATE DRUG RESISTANCE BY MODULATING ECM

CAFs orchestrate tumor promotion and drug resistance by increasing matrix stiffness via augmenting the expression of ECM components such as hyaluronic acid (HA) and collagens^[143]^. Both HA and collagen are known to withstand tensile stress and the activity of collagen receptor, integrin α11β1, is associated with matrix stiffness. It has been reported that in NSCLC, CAFs can promote the stiffness of interstitial collagen by enhancing the expression of integrin α11, leading to tumor progression^[144]^. A recent study demonstrated that collagen secreted by CAFs acts in a paracrine manner to regulate the resistance to microtubule-directed chemotherapeutic drugs through integrinβ1/PI3K/AKT pathway in breast cancer [Figure 2D]^[145]^. The dense number of CAFs was observed to play an important role in desmoplastic reactions in PDAC^[146]^. In addition, the CAFs-derived intense desmoplastic response in fibrotic tumors builds a dense ECM barrier that reduces the delivery of any drug to the tumor cells. In breast cancer patients, a progressively noncompliant fibrotic stroma limits the chemotherapeutic efficiency of doxorubicin^[147]^. Also, based on desmoplastic scores, CAFs have been divided into high desmoplastic CAFs (HD-CAFs) and low desmoplastic CAFs (LD-CAFs). The NSCLC patients with HD-CAFs showed a high collagen matrix remodeling rate which played a critical role in tumor progression via regulation of invasion and growth^[148]^. Likewise, HD-CAFs (alpha-smooth muscle actin positive myofibroblasts) in PDAC are significantly involved in the secretion of type I collagen (Col1) which plays a role in restricting drug delivery and impeding T cell infiltration^[149]^.

Further, CAFs produce metalloproteinases (MMPs) that enhance tumor invasion by matrix remodeling^[150]^. CAFs employ MMP endopeptidases for the degradation of basement membrane (BM) proteins^[151]^. A previous study demonstrated the role of CAFs in BM stretching that facilitates the migration of CAFs and tumor cells into the bloodstream and metastasizes to distant organs. Intriguingly, the alternative CAF-dependent mechanism where BM shows a high tendency of stretching due to low expression of type IV collagen and laminin and rendering the head and neck tumor cells resistant to MMP inhibitors^[152]^. Another study reported that MMPs derived from CAFs are involved in tamoxifen resistance through EGFR and PI3K/AKT pathways in breast cancer [Figure 2D]^[153]^.

In addition to this, CAFs also secrete other factors such as caveolin-1 and podoplanin (PDPN), which are associated with wound responses^[154]^. The expression of a lymphatic vessel marker, PDPN, by stromal CAFs has been reported as a prognostic indicator in different cancer types. For instance, Yoshida *et al.* have demonstrated that lung adenocarcinoma cells, when co-cultured with the PDPN+ CAFs, show greater drug resistance in comparison to normal cells. Similarly, in postoperative recurrence, PDPN+ patients possess a lower treatment response to EGFR-TKIs compared to PDPN- patients, suggesting the implication of PDPN+ CAFs in regulating drug resistance^[155]^. The above information indicates that dense ECM produced by CAFs acts as a mechanical barrier for drug delivery and immune cell infiltration, and it also provides the source for matrix remodeling enzymes and signaling molecules that further impede the efficacy of anticancer therapeutics. Therefore, ECM-depletion strategies might pave the way for the development of next-generation anticancer drugs.

## TARGETING CAFS WITH NATURAL PRODUCTS AND ANALOGS

The utilization of natural products for the treatment of different diseases is indeed cost-effective and minimally invasive^[156-158]^. Numerous anticancer products have been isolated and characterized from natural sources. Apart from directly showing anticancer activity, they also provide leads for developing potent therapeutic drugs^[159]^.

CAFs have emerged as an intriguing therapeutic target in cancer due to their indispensable role. CAFs and cancer cells reciprocally crosstalk to regulate several aspects of cancer progression and several growth factors and cytokines act as a messenger in this crosstalk [Table 1]. Hence, targeting CAFs using natural products will be advantageous for reducing the burden of cancer as well as overcoming deleterious effects caused by drug treatments in cancer patients. Polyphenols present in green tea have potential anticancer activities. Treatment with tea polyphenol, epigallocatechin-3-gallate (EGCG), decreases the serum levels of HGF and VEGF in prostate cancer patients. Since HGF and VEGF are mostly secreted by stromal myofibroblasts in the tumor microenvironment, EGCG can prevent myofibroblast differentiation in prostate cancer^[160]^. Gray *et al.* have demonstrated that combinational treatment of EGCG and another polyphenol, luteolin, synergistically reduces TGF-β-induced myofibroblast differentiation and fibronectin synthesis by impeding ERK and RhoA signaling in prostate fibroblasts^[161]^. CAFs are one of the key contributors in introducing drug resistance to various anticancer chemotherapeutic agents. It was shown that CAFs are involved in acquiring the resistance to cisplatin by expressing Wnt16^[162]^. Quercetin is a member of flavonoids that show antioxidant properties. In this regard, Hu *et al.* have found that quercetin significantly inhibits Wnt16 expression in activated fibroblasts, thereby improving the anticancer effects of cisplatin^[163]^. Various reports have shown that curcumin, a phyto-polyphenol pigment found in spice turmeric, exhibits antioxidant, anti-inflammatory, neuroprotective, and anticancer activities against various cancers^[164]^. In addition, curcumin also regulates the TME of CAFs. An earlier study has shown that curcumin induces DNA damage-independent and safe-senescence in CAFs by upregulating p16. It also decreases the expression of α-SMA and reduces the migration and invasion potentials of CAFs. Furthermore, curcumin abolishes tumorigenic potentials of CAFs by downregulating the expression of IL-6, SDF-1, MMP-2, MMP-9 and TGF-β^[165]^. In the pancreatic cancer model, curcumin suppresses the expression of α-SMA, vimentin, and secretory factors in CAFs, thereby inhibiting EMT and metastasis of cancer cells^[166]^. The above reports indicate that curcumin might have therapeutic potential for impeding the crosstalk between cancer cells and CAFs.

**Table 1:** The interactions between CAFs and cancer cells

**Source cells**	**Factors**	**Recipient cells**	**Biological effect of released factors**	**Affected signaling pathways**	**Reference**
CAFs	CXCL12	Breast cancer	OPN-CAF-derived CXCL12 promotes EMT	ERK1/2 and AKT	[8]
CAFs	CCL5	Ovarian cancer	Cisplatin resistance	STAT3/PI3K/Akt pathway	[81]
CAFs	SDF-1	Lung cancer	Chemoresistance	mir-1/SDF-1/CXCR4/NF-κB/Bcl-xL	[83]
CAFs	IGF-1, HGF	Lung cancer	EMT in NSCLC	IGF1/HGF/ANXA2	[84]
CAFs	IL-6	Ovarian cancer	Paclitaxel resistance	TGFβ/JAK2/STAT3/IL6 pathway	[87]
CAFs	SDF-1	Pancreatic cancer	Gemcitabine resistance	SDF-1/CXCR4/SATB-1 pathway	[88]
CAFs	CCL1	Colorectal cancer	Chemoresistance to 5-FU and paclitaxel	TGF-β/NF-κB pathway	[89]
CAFs	IL-8	Gastric cancer	Cisplatin resistance	NF-κB pathway	[91]
Breast cancer	TGF-β1	CAFs	5-FU and tamoxifen (TAM) resistance	p44/42 MAPK pathway	[94]
CAFs	CXCL12	Ovarian cancer	EMT and cisplatin resistance	CXCR4/Wnt/β-catenin pathway	[95]
CAFs	miR-24-3p	Colon cancer	Resistance to methotrexate	CDX2/HEPH axis	[99]
CAFs	miR-148b-3p	Bladder cancer	EMT, metastasis, and drug resistance	Wnt/β-catenin pathway	[102]
CAFs	IL-17A	Cancer-initiatingcells	Resistance to chemotherapies	IL-17A signaling pathway	[106]
CSCs	SHH	CAFs	CSCs expansion	Wnt/β-catenin signaling pathway	[109]
CAFs	TGF-β2	CSCs	Chemoresistance and stemness of CSCs	HIF-1α/TGF-β2-GLI2 pathway	[112]
CAFs	H19	CRC	Elevates stemness in CRCs	miR-675-IGFR signaling circuit & β-catenin pathway	[114]

Fraxinellone (FRA) is a member of the limonoids family. Several studies have reported the medicinal properties of FRA, including neuroprotective, antifibrotic, anti-inflammatory, and antitumor functions^[167]^. An earlier study has reported that FRA regulates TGF-β signaling in fibrotic liver disease^[168]^, which hints therapeutic potential of FRA in treating cancer, as both are characterized by the accumulation of myofibroblasts. A recent report demonstrated that FRA-loaded nanoparticle inhibits the CAF phenotype by impeding TGF-β signaling in PDAC^[169]^. Mangostin (MG) is a xanthone and exhibits several medicinal properties such as antibacterial, antifungal, antioxidant, anti-inflammatory, anticancer, and cardioprotective effects^[170]^. Studies have demonstrated that MG displays antitumor activities by inducing apoptosis and inhibiting angiogenesis, ECM modification, and EMT^[171]^. A study has recently demonstrated MG’s effect in regulating the tumor stroma. A nano-formulated MG suppresses TGF-β/Smad signaling leading to CAF inactivation and ECM reduction in pancreatic cancer^[172]^.

Cyclopamine is a steroid alkaloid and the first small-molecule inhibitor of the Hh signaling pathway^[173]^. Several reports show that Hh signaling displays a critical role in proliferation and tumor-promoting functions indicating the potential of cyclopamine to reprogram CAFs^[174,175]^.

Co-delivery of cyclopamine and paclitaxel nanoparticles in pancreatic cancer modulates tumor stroma by disrupting cancer-stroma crosstalk and reducing ECM stiffness^[174]^. Chrysin is classified as a member of the flavonoids, which exerts multiple biological effects including antidiabetic, antioxidant, hepatoprotective, anti-inflammatory, and anticancer activities^[176]^. Chrysin induces apoptosis in colorectal and gastric cancer cells^[177,178]^. A synthetic analog of chrysin named 8-bromo-7-methoxy chrysin inhibits the activation of hepatic stellate cells to CAFs, thereby reducing the stemness of cancer cells by impeding IL-6 and HGF signaling^[179]^. We have listed several natural or synthetic drugs for targeting CAFs in Table 2.

**Table 2:** Drugs targeting CAFs for management of cancer

**Drug** **natural**	**Type of cancer**	**Target/Interference with**		**Mechanism**	**Reference**
		**CAFs**	**CAFs functions**		
EGCG	Colorectal		↓Glycolytic activity	↓PFK	[180]
Conophylline	HCC	↓α-SMA	↓IL6, IL8, CCL2, angiogenin, OPN	↓GPR68	[181]
α-mangostin	Pancreatic	↓αSMA/FAP/fibronectin	↓fibronectin/collagen	↓TGF-β pathway/Smad	[172]
Fraxinellone	Pancreatic	↓αSMA/FAP/fibronectin	-	↓TGF-β pathway	[169]
Triptonide	Gastric	-	↓IL-6, ↑TIMP2	↓MiR-301a ↑MiR-149	[182]
Chrysin	Liver	-	↓IL-6, HGF	-	[179]
Paeoniflorin	Gastric	-	↓IL-6	↑MicroRNA149	[183]
Resveratrol	CCA	-	↓IL-6	-	[184]
Minnelide	Pancreatic	↓α-SMA	↓Collagen/fibronectin/periostin/hyaluronan/ MMP2/MMP9	↓TGF-β RAR/RXR pathway	[185]
Cyclopamine	Pancreatic	-	↓LOX/hyaluronan	↓Hh pathway	[186]
Polyphyllin I	Gastric	↓FAP	↓HGF	-	[187]
Curcumin	Pancreatic	-	↑E-cadherin, ↓vimentin	-	[166]
Astragaloside IV	Gastric	-	↓M-CSF, ↑TIMP2	↑microRNA-214 ↓microRNA-301a	[188]
**Synthetic drugs**					
Ursolic acid	PTC	-	↓CXCR4, CXCR7	-	[189]
CFH/OM-L	Hepatic	-	↑E-cadherin, ↓vimentin, N-cadherin, snail protein	-	[190]
Nintedanib	Hepatic	↓α-SMA	IL-6, IL-8	-	[191]
BTZ and PST		-	-	↑Caspase-3 mediated apoptosis	[192]
**Drugs under clinical trial**					
JNJ-42756493	NSCLC, Urothelial, Esophageal	↓FGFR ↓TK	-	-	[193]
Plerixafor	Pancreatic, Ovarian and Colorectal	-	↓CXCR4	-	[194]
PEGPH20 and MK-3475	PDAC	-	↓Hyalouronan	-	[195]
AT13148	Advanced solid tumors	-	↓ROCK	-	[196]
IPI-926 and Gemcitabine	Pancreatic	-	-	↓Hh pathway	[197]

α-SMA: A-smooth muscle actin; BTZ: bortezomib; CCA: cholangiocarcinoma; CCL2: C-C motif chemokine ligand 2; CFH/OM-L: CFH peptide (CFHKHKSPALSPVGGG)-decorated liposomal oxymatrine; EGCG: epigallocatechin-3-gallate; FAP: fibroblast activation protein alpha; HCC: hepatocellular carcinoma; Hh: hedgehog; MMP2: matrix metalloproteinase 2; OPN: osteopontin; PFK: phosphofructokinase; PTC: papillary thyroid carcinoma; PST: Panobinostat; TIMP2: tissue inhibitor of metalloproteinase 2.

## COMPOUNDS UNDER CLINICAL TRIAL

There are several compounds under clinical trials for targeting the CAFs or CAF-mediated effects for the management of cancer^[198]^. Notably, losartan, a small molecular inhibitor, sold under the brand name Cozaar, is used for the treatment of diabetic kidney disease, heart failure, and left ventricular enlargement. It inhibits the angiotensin receptor by blocking binding of angiotensin II. It suppresses collagen and hyaluronan levels, which are known to be synthesized by CAFs, and it is currently under clinical trial^[199]^. Defactinib is a small molecular inhibitor of FAK, available under brand names, VS-6063 and PF-04554878. It is under phase II clinical trial for the treatment of patients with KRAS-mutant NSCLC and is known to target downstream signaling of integrins and interfere with CAF actions^[200]^. Vitamin D receptor agonist, paricalcitol, is under phase I and II studies to examine the benefit of it in combination with gemcitabine and nab-paclitaxel for the treatment of pancreatic cancer as it is known to normalize pancreatic stellate cells^[201]^. Galunisertib is a pharmacological small molecule inhibitor of the TGF-β signaling. Treatment with Galunisertib interferes with TGF-β signaling induced activation of CAFs and immunosuppression. Studies of phase I, and phase II trials are underway to compare the overall survival (OS) of patients with pancreatic cancer after the treatment with a combination of Galunisertib and gemcitabine as compared to gemcitabine alone^[202,203]^. IPI-926 (saridegib) and vismodegib are small molecular inhibitors that target Hh signaling and reduce CAF activation, and are under clinical trials^[204,205]^. Several compounds for targeting CAFs are under clinical trials [Table 2].

## CONCLUSIONS AND FUTURE DIRECTIONS

Stromal fibroblasts constitute a major component of tumor microenvironment. Fibroblasts can thrive in severe adverse conditions because of their intrinsic survival programs and cellular plasticity. Due to this ability, they can withstand insults from anticancer regimens. Simultaneously, these cells activate cell resistance programs in cancer cells in response to inhibitory effects caused by the treatment modalities. Therefore, it is very important to understand the intrinsic survival programs and cellular plasticity that endure these cells from chemotherapy insults. Since the reciprocal interactions between the cancer cells and CAFs through soluble factors play a crucial role in orchestrating drug resistance programs, it is critical to profile CAF-derived secretome in response to chemotherapy to identify druggable targets. The CAF-mediated direct cell-cell and cell-matrix interactions also shape drug resistance in cancer cells. Hence, investigating the ECM-remodeling and changes in cell adhesion molecules triggered during the development of drug resistance might provide insights into target proteins. Moreover, the double-edged sword effect of CAF signaling has been well recognized in tumor progression and drug resistance. Dissecting CAF-signaling and its function enables comprehensive identification of signaling pathways required to instigate drug resistance programs and facilitates targeting drug-resistant inducing cues and sparing the inhibitory cues.

## DECLARATIONS

### Copyright

© The Author(s) 2023.
